# TMEM132A, a Novel Wnt Signaling Pathway Regulator Through Wntless (WLS) Interaction

**DOI:** 10.3389/fcell.2020.599890

**Published:** 2020-11-26

**Authors:** Binbin Li, Lee A. Niswander

**Affiliations:** Department of Molecular, Cellular, and Developmental Biology, University of Colorado Boulder, Boulder, CO, United States

**Keywords:** transmembrane protein 132A (TMEM132A), Wntless (WLS), Wnt signaling pathway, Wnt ligand, intracellular trafficking

## Abstract

Wnt signaling pathway plays indispensable roles in embryonic development and adult tissue homeostasis. However, the regulatory mechanisms involved in Wnt ligand trafficking within and secretion from the signal sending cells is still relatively uncharacterized. Here, we discover a novel regulator of Wnt signaling pathway called transmembrane protein 132A (TMEM132A). Our evidence shows a physical and functional interaction of TMEM132A with the Wnt ligand transporting protein Wntless (WLS). We show that TMEM132A stabilizes Wnt ligand, enhances WLS–Wnt ligand interaction, and activates the Wnt signaling pathway. Our results shed new light on the cellular mechanism underlying the fundamental aspect of WNT secretion from Wnt signal sending cells.

## Introduction

Embryonic development and adult tissue homeostasis depend on the Wnt signaling pathway. Abnormal Wnt signal transduction is associated with multiple birth malformations and human diseases (Freese et al., 2010; Yang, 2012). In WNT secreting cells, nascent Wnt ligands are post-translationally modified in the endoplasmic reticulum (ER), shuttled to the Golgi apparatus and then transferred to the plasma membrane carried by an evolutionally conserved eight-pass transmembrane protein Wntless (WLS, also known as GPR177), the ortholog of Mig-14 in *Caenorhabditis elegans* and Evi in *Drosophila*, for exocytosis (Banziger et al., 2006; Bartscherer et al., 2006). WLS is indispensable and exclusively required for the secretion of virtually all Wnt ligands across animals (Ching and Nusse, 2006; Sun et al., 2017). After releasing Wnt ligand from the secreting cell to the extracellular space to trigger Wnt pathway activation in neighboring cells, WLS undergoes Clathrin-dependent endocytosis and SNX3-dependent retrograde trafficking to the Golgi and then ER for recycling (Coombs et al., 2010; Gasnereau et al., 2011; Harterink et al., 2011; Yu et al., 2014; McGough et al., 2018; Brown et al., 2020). Recycling allows WLS to be reutilized for WNT binding to continue a robust cycle of WNT secretion. Only a few factors required for WLS-WNT ligand trafficking have been identified, such as P24 family proteins, the small GTPases RAB8A and SAR1, and SAR1 specific guanine nucleotide-exchange factor (GEF) SEC12 (Buechling et al., 2011; Port et al., 2011; Das et al., 2015; Sun et al., 2017). The importance of WLS-Wnt ligand trafficking in tissue development, homeostasis and disease makes an understanding of its regulation a pressing task to be solved.

Here, we identify a novel role for the single-pass transmembrane protein 132A (TMEM132A) in regulating Wnt signaling pathway through interaction with WLS. TMEM132A belongs to the TMEM132 family composed of five members (TMEM132A-E). The structure and molecular function of this protein family remain poorly understood and under-investigated. TMEM132A was originally identified as a binding protein for the ER resident chaperone 78-kDa glucose-regulated protein (GRP78, also called as BIP or HSPA5) in rat brain cells, and thus is also named GBP and HSPA5 binding protein 1 (HSPA5BP1) (Oh-hashi et al., 2003). TMEM132A is also reported to regulate the expression of cAMP-induced glial fibrillary acidic protein in rat glioblastoma cells (Oh-hashi et al., 2006). Robust TMEM132A expression was found in rat embryonic brain predominantly and several other tissues, suggesting a functional role in the developing brain and perhaps a role in preventing neurodegeneration (Oh-hashi et al., 2003). Knockdown of TMEM132A in Neuro-2a cells increases serum starvation-induced ER stress related apoptosis (Oh-hashi et al., 2010). Taken together with the evidence for interaction with the ER chaperone GRP78, it is suggested that TMEM132A might be an important factor for cell survival.

In this study, we identify a new protein interaction between TMEM132A and the Wnt ligand transporting protein WLS. We show that depletion of TMEM132A results in destabilized Wnt ligand, and weakened WLS–Wnt ligand interaction. This leads to repressed Wnt signaling pathway. Our results broaden the current understanding of the indispensable Wnt signal transduction pathway in the context of the signal sending cells.

## Results

### TMEM132A Is a Self-Interacting Type I Transmembrane Protein

**FIGURE 1:**
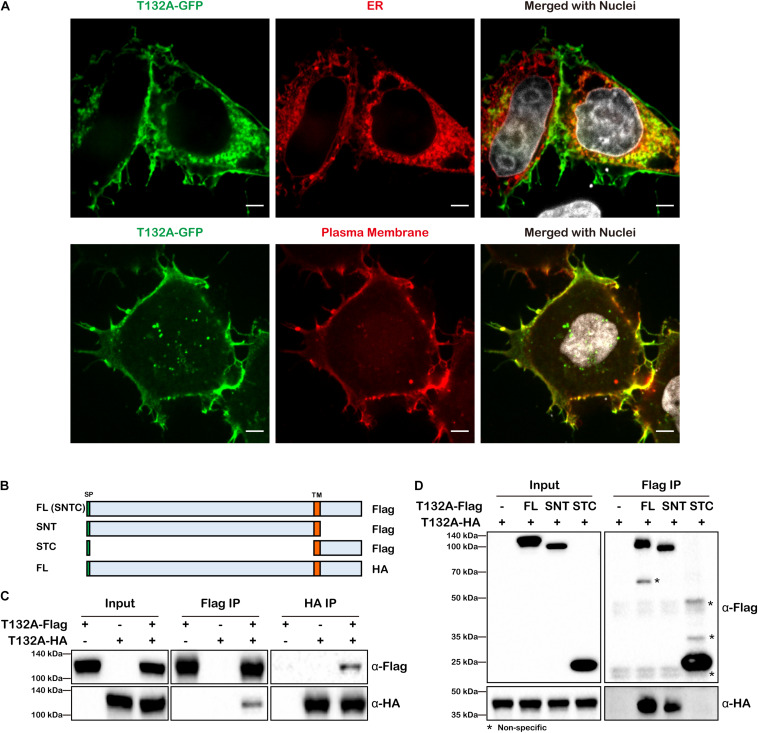
TMEM132A type I transmembrane protein localizes to the plasma membrane and ER and interacts with itself. **(A)** HeLa cells transiently expressing FL T132A-GFP, ER tranker of ER-RFP (upper panel) or plasma membrane tracker of PM-RFP (lower panel) were fixed, nuclei counterstained with Hoechst, and imaged under 100× oil objective lens. Fluorescent signals were pseudo-colored in green, red or gray as indicated. Scale bars: 5 μm. **(B)** Schematic representation of FL T132A-Flag consisting of Signal peptide (SP), N-terminus, Transmembrane (TM) domain and C-terminus (abbreviated as SNTC), truncated T132A-Flag of SNT or STC, and FL HA-tagged TMEM132A (T132A-HA). **(C)** Co-IP between FL T132A-Flag and T132A-HA in HEK 293T cells. **(D)** Co-IP of FL T132A-HA by FL and SNT, but not by STC of T132A-Flag in HEK 293T cells. * indicates non-specific bands.

In on-going studies of neural tube defects, we became interested in TMEM132A, loss of which causes spina bifida in mice^1^. As little is known as to TMEM132A function, the following studies were conducted to identify potential TMEM132A interacting proteins to gain insight into possible mechanisms by which TMEM132A regulates cellular activity. Sequence analysis shows TMEM132A is type I single-pass transmembrane protein, with a longer *N*-terminal domain that faces inside the ER lumen and which subsequently would be on the outside of the plasma membrane. To assess its subcellular localization, *C*-terminal turbo GFP-tagged full-length (FL) TMEM132A (T132A-GFP) was transfected into HeLa cells along with marker for the ER or the plasma membrane and visualized by confocal microscopy. Previous studies in COS-7 cells with GFP-tagged TMEM132A showed overlap with the fluorescent probe (BODIPY)-labeled Brefeldin A (BFA) in the ER apparatus (Oh-hashi et al., 2003, 2012), although BFA is an ER to Golgi transport inhibitor and would be expected to cause protein accumulation in the ER (Chardin and McCormick, 1999). Even so, in untreated cells we found that TMEM132A colocalizes with the ER marker (Figure 1A, upper panel). It was also reported that TMEM132A localizes in the plasma membrane (Oh-hashi et al., 2012). Indeed, in almost all cells we noticed robust signal at the plasma membrane, in particular the pseudopodium and cell–cell junction area. In addition, TMEM132A was also found in some vesicle-like organelles (Figure 1A, lower panel).

As many transmembrane proteins, such as cell adhesion molecules and signal receptors, form homodimers or oligomers, we tested whether TMEM132A also interacts with itself. Co-immunoprecipitation (co-IP) experiment revealed self-association of differentially tagged FL TMEM132A constructs expressed in HEK 293T cells (Figures 1B,C). To map the domain of self-association, *N*-terminal (SNT) and *C*-terminal (STC) constructs were created, both containing the signal peptide (SP) and transmembrane (TM) domain (Figure 1B). Deletion of the *N*-terminus, but not the *C*-terminus, abolishes the TMEM132A self-interaction property (Figure 1D). This suggests that TMEM132A likely functions as a dimer or oligomer and that interaction occurs through the *N*-terminal domain.

### TMEM132A Interacts With the Wnt Ligand Trafficking Protein WLS

**FIGURE 2:**
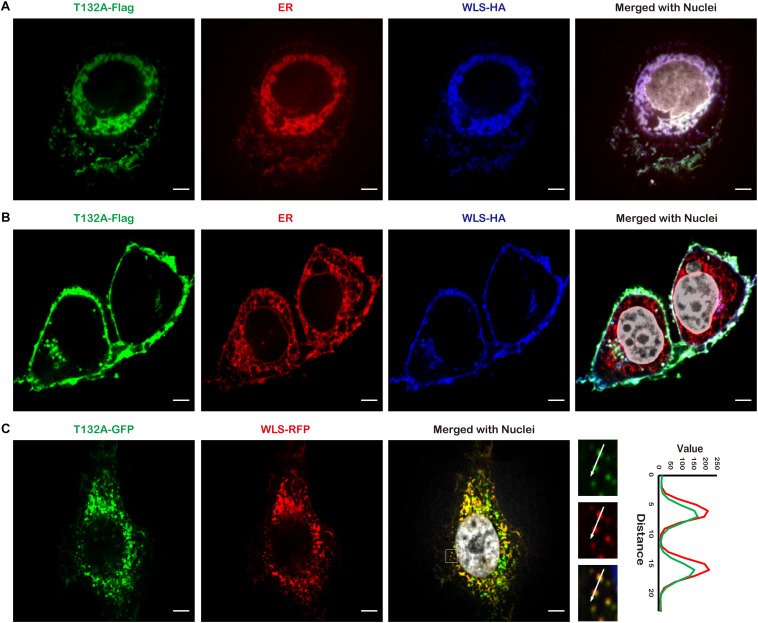
TMEM132A colocalizes with WLS. **(A,B)** HeLa cells co-transfected with FL T132A-Flag, FL WLS-HA, and ER-RFP were fixed and immunostained, nuclei counterstained with Hoechst. **(C)** HeLa cells co-transfected with FL T132A-GFP and FL WLS-RFP were fixed and nuclei counterstained with Hoechst. The fluorescence intensity in the boxed area was quantified along the indicated white arrow for both channels. Cells were imaged under 100× oil objective lens. Fluorescent signals were pseudo-colored in green, red, blue, or gray as indicated. Scale bars: 5 μm.

In an effort to identify candidate TMEM132A interacting partners, we utilized HEK 293T cells with a stable integration of TMEM132A expression cassette, tandem affinity purification (TAP), filter-aided sample preparation (FASP) and mass spectrometry (details on methodology and identified proteins are included in Supplementary Materials). A major interacting protein identified is WLS. To visualize whether TMEM132A and WLS colocalize in cells, HeLa cells were co-transfected with Flag-tagged FL TMEM132A (T132A-Flag), HA-tagged FL WLS (WLS-HA), and turbo RFP-tagged ER marker (ER-RFP), immunostained with tag-specific antibodies, and imaged by confocal microscopy. Consistent with reports that WLS predominantly localizes to the ER apparatus (Coombs et al., 2010; Yu et al., 2014), Figure 2A shows that the TMEM132A and WLS signals largely overlap in the ER apparatus. Moreover, in a predominant number of cells, TMEM132A and WLS colocalize on the cell surface (Figure 2B), similar to the distribution of TMEM132A at the plasma membrane shown in Figure 1. Furthermore, as shown in Figure 2C, T132A-GFP and turbo RFP-tagged FL WLS (WLS-RFP) colocalize in numerous vesicle-like organelles. These data indicate that TMEM132A colocalizes with WLS along its exocytic transport pathway from the ER, into vesicles, and to the plasma membrane, suggesting that TMEM132A facilitates WLS transport from the ER apparatus to the plasma membrane.

**FIGURE 3:**
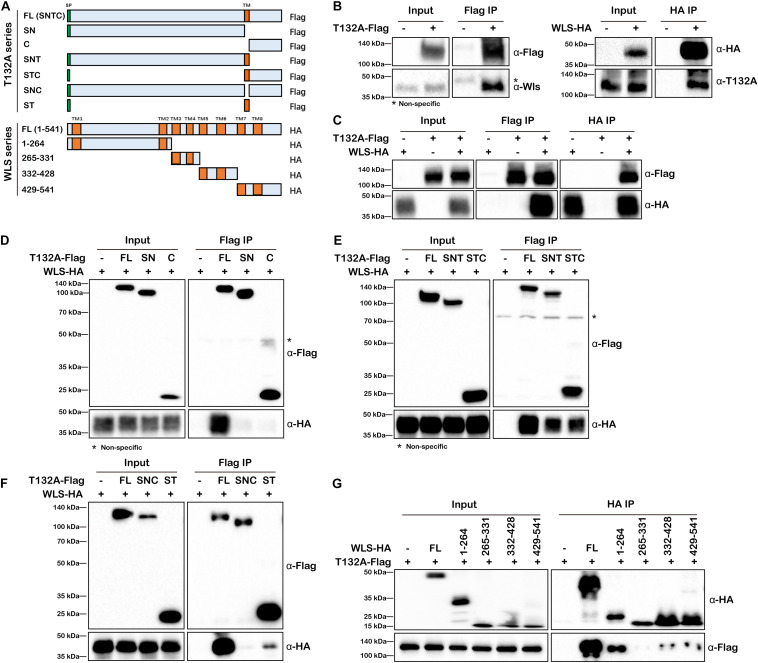
TMEM132A and WLS interact with each other. **(A)** Schematic representation of FL and truncated constructs of T132A-Flag and WLS-HA. **(B)** Co-IP of endogenous WLS by FL T132A-Flag (left), and co-IP of endogenous TMEM132A by FL WLS-HA (right) in HEK 293T cells. **(C)** Co-IP between FL T132A-Flag and FL WLS-HA in HEK 293T cells. **(D)** Co-IP of WLS-HA by FL, but not SN or C expression constructs lacking TM domain of T132A-Flag in HEK 293T cells. **(E)** Co-IP of WLS-HA by FL, SNT and STC of T132A-Flag in HEK 293T cells. **(F)** Co-IP of WLS-HA by FL and ST of T132A-Flag but not by the SNC construct lacking TM domain of T132A-Flag in HEK 293T cells. **(G)** Co-IP showing strong interaction of FL T132A-Flag with FL and truncation 1-264 of WLS-HA, and weak interaction with truncations 332–428 and 429–541 of WLS-HA in HEK 293T cells. * indicates non-specific band.

To validate TMEM132A–WLS interaction, we performed co-IP experiments. As there is no available commercial antibody against TMEM132A or WLS suitable for IP, we first tested the interaction using overexpressed FL T132A-Flag to co-IP endogenous WLS in HEK 293T cells (Figure 3B, left panels). We then used the same strategy to show that endogenous TMEM132A can also be pulled-down by overexpressed FL WLS-HA (Figure 3B, right panels). In addition, overexpressed T132A-Flag and WLS-HA can be co-immunoprecipitated by each other, as shown in Figure 3C.

In order to map the responsible domain or motif that mediates TMEM132A–WLS interaction, a series of truncations of the two proteins were constructed (Figure 3A) for co-IP analysis and these Flag or HA-tagged expression constructs were transfected into HEK 293T cells as noted, and subsequently whole cell lysates prepared. Neither the SN nor the C truncation of TMEM132A interacts with WLS efficiently (Figure 3D), but the addition of the TM domain restores the interaction with WLS for both (the SNT & STC lanes in Figure 3E). A construct in which only the TM domain is removed from the FL TMEM132A (SNC) also does not interact with WLS (Figure 3F). This could reflect mis-localization of those proteins without TM domain. However, a construct with only the signal peptide and TM domain can interact with FL WLS (ST lane in Figure 3F). These results demonstrate that the TM domain of TMEM132A is the crucial compartment for the interaction with WLS. This is similar to reports that a TM domain can serve as interacting domain. For example, Gerlach et al. (2018) demonstrated that TMEM59 interacts with Frizzled (FZD), a cell surface co-receptor for Wnt ligand, via its TM domain. Our mass spectrometry data did not identify FZD nor many other TM proteins, suggesting a more specific interaction of the TMEM132A TM domain with WLS. We then performed the reciprocal mapping to determine the domain of WLS that interacts with TMEM132A using a similar strategy. WLS has eight TM domains, so WLS was divided into four constructs and each contains two TM domains (Figure 3A). Previous studies found that amino acids (aa) 100-232 make up the Wnt ligand binding domain (Sun et al., 2017). A construct with WLS aa 1–264 including TM domains 1 and 2 and Wnt ligand binding domain shows the strongest interaction with TMEM132A (Figure 3G). Weak interaction with TMEM132A was also found for WLS aa 332–428 and aa 429–541 truncations, indicating that additional TMEM132A-interacting motifs might exist in these regions as well.

### Depletion of TMEM132A Reduces Cytoplasmic and Secreted Wnt Ligand Levels

**FIGURE 4:**
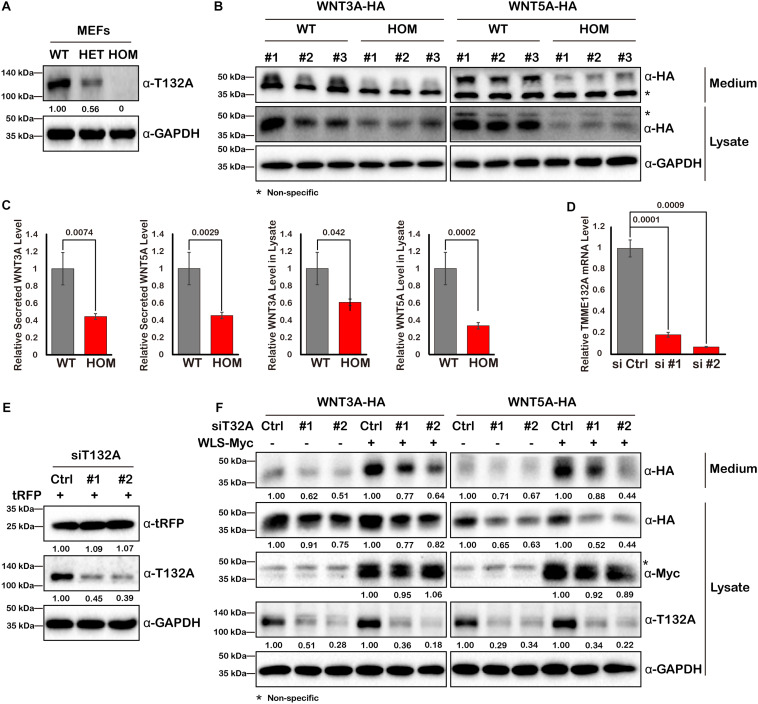
WNT3A and WNT5A protein levels are decreased upon loss of TMEM132A. **(A)** MEFs were isolated from crosses of *Tmem132a^+/–^* male and female mice to obtain WT, HET, and HOM embryonic fibroblasts, and TMEM132A protein levels were determined by Western blot. GAPDH served as an internal control. Relative band intensity is given below corresponding panel (normalized to GADPH). **(B)** MEFs were transfected with FL WNT3A-HA or WNT5A-HA, 48 hours later the culture medium was collected and the cells were lysed for Western blot with GAPDH as an internal control. Both intracellular and secreted levels of WNT3A or WNT5A were decreased by loss of TMEM132A, and band intensity was quantified (normalized to GADPH) in **(C)**. **(D)** TMEM132A specific siRNAs (#1 and #2) were used to knockdown TMEM132A compared to scramble siRNA (Ctrl) in HEK 293T cells and the efficiency was evaluated by real-time quantitative PCR 48 hours post transfection. *GAPDH* served as an internal control. **(E)** HEK 293T cells were co-transfected with scramble or TMEM132A specific siRNAs, and pCMV6-AC-RFP as a transfection control, and cell lysates collected 24 hours post transfection for Western blot. Relative band intensity is given below corresponding panel (normalized to GADPH). **(F)** HEK 293T cells were co-transfected with scramble or TMEM132A specific siRNAs, FL WNT3A-HA or WNT5A-HA, and FL WLS-Myc in indicated groups, and the culture medium and cell lysate were collected 48 hours post transfection for Western blot. Knockdown of TMEM132A results in decreased cytoplasmic and secreted WNT3A or WNT5A protein levels. Relative band intensity is given below corresponding panel (normalized to GADPH). * indicates non-specific band.

Since WLS is exclusively required for Wnt ligand trafficking, we next assessed the impact of TMEM132A on Wnt ligand protein level. Mouse embryonic fibroblast cells (MEFs) were isolated from wild type or a deletion allele of *Tmem132a* mouse embryos. Western blot confirmed that TMEM132A protein was disrupted as seen in heterozygous (HET) and homozygous (HOM) mutant MEFs (Figure 4A). Wild type and mutant MEFs were transfected with the canonical Wnt/β-Catenin signaling pathway ligand WNT3A and the non-canonical Wnt/planar cell polarity (PCP) signaling pathway ligand WNT5A. Forty-eight hours post transfection, the cell culture medium was collected and clarified by centrifuge for the study of Wnt ligand secretion, and the corresponding cell lysate was prepared for analysis of total Wnt ligand protein level within the cells. This shows that both the cytoplasmic and secreted WNT3A and WNT5A protein levels are significantly lower in TMEM132A knockout MEFs, compared to wild type cells (Figures 4B,C).

As another method to confirm a specific, rather than perhaps indirect effect, of loss of TMEM132A on Wnt ligand levels, two different small interfering RNAs (siRNAs) were used to knockdown TMEM132A expression in HEK 293T cells. The silencing efficiency was assessed by real-time quantitative PCR (Figure 4D) and Western blot (Figure 4E). Coincident with the previous finding, repression of TMEM132A results in lower levels of cytoplasmic and secreted WNT3A and WNT5A, compared to the scramble control siRNA group (Figure 4F). The transfection efficiency was comparable between scramble siRNA and siTMEM132A cells based on co-transfected turbo RFP (tRFP) (Figure 4E). To boost Wnt ligand secretion, WLS was co-transfected with the siRNAs. The same trend was observed in that knockdown of TMEM132A reduces cytoplasmic and secreted WNT3A and WNT5A levels (Figure 4F). Taken together, loss of TMEM132A leads to lower Wnt ligand protein levels. However, this experiment does not distinguish whether the reduction in secreted Wnt ligand results from a secretion defect or reflects the reduced Wnt ligand pool in the cytoplasm.

### TMEM132A Positively Regulates the Wnt Signaling Pathway

**FIGURE 5:**
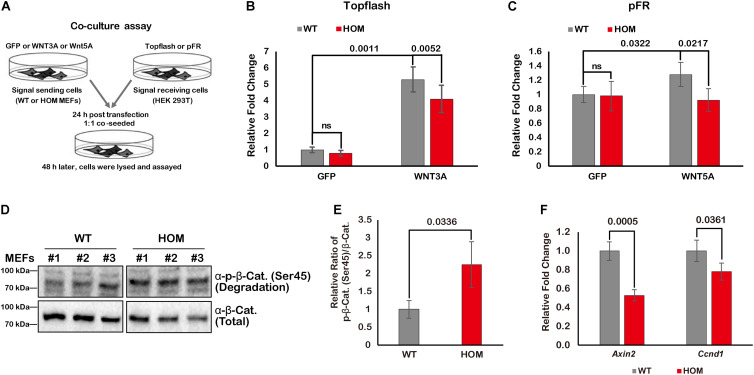
TMEM132A positively regulates Wnt signaling pathway. **(A)** Schematic representation of co-culture and dual-luciferase assays. Topflash firefly luciferase reporter was used to measure canonical Wnt/β-Catenin signaling pathway activation in **(B)**, and pFR firefly luciferase reporter accompanied by pFA2-c-Jun were used to evaluate non-canonical Wnt/PCP signaling pathway activation in **(C)**. Constitutively expressed Renilla reniformis luciferase served as an internal control (*n* = 3 biological replicates; *p*-value was calculated by Student’s *t*-test). **(D)** The levels of phospho-β-Catenin (Ser45) and total β-Catenin were determined by Western blot in whole cell lysate of untransfected wild type and TMEM132A mutant MEFs. **(E)** The ratio of phospho-β-Catenin (Ser45)/total β-Catenin was calculated from the band intensity in panel **(D)**. The result shows β-Catenin becomes unstable by loss of TMEM132A (*n* = 3 biological replicates; *p*-value was calculated by Student’s *t*-test). **(F)** Real-time quantitative PCR was performed and shows relative transcription level of canonical Wnt/β-Catenin signaling pathway target genes *Axin2* and *Ccnd1* are downregulated by loss of TMEM132A (*n* = 3 biological replicates; *p*-value was calculated by Student’s *t*-test). *GAPDH* served as an internal control.

To test whether TMEM132A is required for activation of the canonical Wnt/β-Catenin and Wnt/PCP signaling pathways, we used a co-culture and dual-luciferase reporter assay system (Banziger et al., 2006). Wild type or TMEM132A mutant MEFs were transfected with turbo GFP (negative control) or WNT3A or WNT5A to serve as signal sending or producer cells. HEK 293T cells were transfected with Wnt signaling reporters to serve as the signal receiving or responder cells. Both groups of cells were then trypsinized, counted, and co-seeded with equivalent numbers, and after further culture were subjected to dual-luciferase assay (Figure 5A). Canonical Wnt/β-Catenin signaling pathway is commonly assessed using a Topflash Luciferase reporter which harbors multimerized consensus TCF/LEF binding elements stimulated in conjunction with β-Catenin in the nucleus to drive firefly luciferase expression. As shown in Figure 5B, effective activation of canonical Wnt signaling pathway in signal receiving cells was achieved when signal sending wild type MEFs were transfected with WNT3A ligand compared to turbo GFP. However, loss of TMEM132A in HOM MEFs significantly diminishes the ability of these cells to induce reporter expression. Non-canonical Wnt signaling pathway is more difficult to assess but the reporter based on JNK-c-Jun cascade-dependent activation have been described (Shi et al., 2012, 2018). Here we used the pFR Luciferase reporter harboring activated c-Jun responsive motif in the promoter region of firefly luciferase along with the c-Jun expressing plasmid pFA2-c-Jun (Shi et al., 2012, 2018). As shown in Figure 5C, compared to turbo GFP control, there was modest induction of the reporter in signal receiving cells exposed to WNT5A transfected wild type MEFs but no induction when exposed to WNT5A transfected mutant MEFs. These results indicate that loss of TMEM132A reduces the amount of Wnt ligand to act on receiving cells.

We next further dissected the potential function of TMEM132A relative to the canonical Wnt/β-Catenin signaling pathway, given its output is easier to evaluate than Wnt/PCP signaling pathway. β-Catenin is a core factor in canonical Wnt signal transduction. Upon reception of the Wnt signal, β-Catenin is released from the AXIN/APC destruction complex and enters into the nucleus where it acts with TCF/LEF transcription factors to activate target genes such as *Axin2* and *Cyclin D1* (*Ccnd1*) (Yang, 2012). In the absence of Wnt ligand binding with cell surface receptors on signal receiving cells, β-Catenin is specifically post-translationally modified (e.g., phosphorylation at Ser45 residue; Amit et al., 2002; Daugherty and Gottardi, 2007) and degraded through the AXIN/APC complex (Clevers and Nusse, 2012). In TMEM132A knockout MEFs (untransfected and without any additional treatment), the phosphorylation level of endogenous β-Catenin at Ser45 is dramatically increased and total β-Catenin protein level is decreased relative to wild type MEFs (Figure 5D). Quantification of the ratio of phosphorylated to total β-Catenin ratio shows that β-Catenin phosphorylation is significantly higher in TMEM132A knockout MEFs versus wild type cells (Figure 5E). This indicates that β-Catenin becomes unstable as a result of the TMEM132A depletion. As further evidence of a defect in Wnt signal transduction, the expression of direct transcriptional targets of the canonical Wnt signaling pathway, *Axin2* and *Ccnd1*, were assessed and found to be repressed in TMEM132A mutant MEFs (Figure 5F). These results demonstrate that TMEM132A is required for efficient induction of the Wnt signaling pathway.

### TMEM132A Increases the Stability of Wnt Ligand Cargo and Its Binding With WLS

**FIGURE 6:**
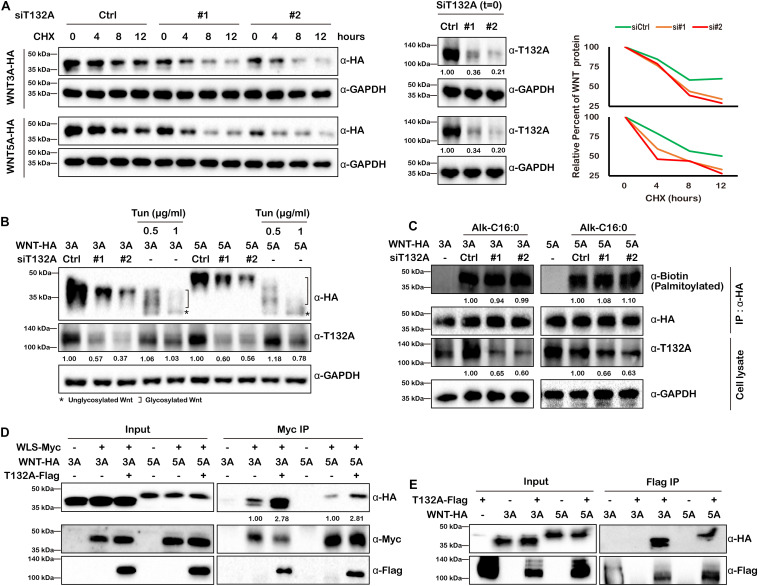
TMEM132A stabilizes Wnt ligands and increases their binding with WLS. **(A)** Loss of TMEM132A destabilizes WNT protein levels. (Left panel) Scramble or TMEM132A siRNAs were transfected into HEK 293T cells followed by transfection with FL WNT3A or WNT5A-HA. After 24 hours the cells were treated with 100 μg/ml CHX and the protein levels of WNT3A or WNR5A were evaluated at the indicted time periods by Western blot. (Middle panel) TMEM132A knockdown efficiency before CHX treatment (*t* = 0). GAPDH served as an internal control. (Right panel) Relative WNT3A (upper panel) or WNT5A protein (lower panel) remained after CHX treatment was calculated by measuring the Western blot band grayscale. **(B)** WNT glycosylation is not altered by TMEM132A depletion. HEK 293T cells were co-transfected with TMEM132A siRNAs and WNT3A or WNT5A-HA, and the glycosylation status of WNT3A or WNT5A was determined by Western blot. Tun at the indicated concentrations was used to block global glycosylation as a positive control. Relative band intensity is given below corresponding panel (normalized to GADPH). **(C)** WNT palmitoylation is not altered by TMEM132A depletion. HEK 293T cells co-transfected with scramble or TMEM132A specific siRNAs and FL WNT3A or WNT5A-HA were labeled with 100 μM palmitic acid alkyne (Alk-C16:0), lysed and subjected to IP and on-bead click chemistry reaction in the presence of biotin azide plus to determine WNT3A/5A palmitoylation level by Western blot. TMEM132A knockdown efficiency is shown, with GAPDH as an internal control. Relative band intensity is given below corresponding panel (normalized to GADPH except the α-Biotin panel which is normalized to α-HA signal). **(D)** Co-IP was performed to determine the interaction between WLS and WNT3A or WNT5A in the presence or absence of overexpressed TMEM132A in HEK 293T cells. The result shows enhanced WLS-WNT3A or WNT5A interaction by addition of TMEM132A. Relative band intensity is given below corresponding panel (normalized to α-Myc signal) **(E)** Co-IP of FL WNT3A-HA and WNT5A-HA by FL T132A-Flag in HEK 293T cells.

Our finding that TMEM132A depletion decreases Wnt ligand protein levels within the cell and in the culture medium (Figures 4B,C,F) raises the possibility that Wnt ligand stability might be affected by loss of TMEM132A. Given that a constitutive CMV promoter was used to drive the expression of WNT3A and WNT5A, we can rule out the possibility of a transcriptional effect of TMEM132A on transfected Wnt ligand. To test whether Wnt ligand stability is affected by loss of TMEM132A, we transfected HEK 293T cells with scrambled siRNA or TMEM132A specific siRNAs and with HA-tagged WNT3A (WNT3A-HA) or WNT5A (WNT5A-HA), then twenty-four hours later added cycloheximide (CHX) to block protein synthesis, and subsequently assessed the level of WNT3A and WNT5A that remained within cells over time. As shown in Figure 6A, WNT3A and WNT5A levels significantly decreased over time when TMEM132A was silenced, indicating increased protein degradation.

The active form of Wnt ligand consists of post-translational modifications such as glycosylation and palmitoylation (Komekado et al., 2007; Kurayoshi et al., 2007). Wnt ligands are initially *N*-glycosylated (at Asn87 and Asn298 sites of WNT3A) mediated by the oligosaccharyl transferase (OST) complex. Dysregulated glycosylation could lead to misfolding of synthesized protein in the ER and induce unfolded protein response (UPR) and ER-associated degradation (ERAD) (Ruggiano et al., 2014). However, the glycosylation status of both WNT3A and WNT5A were similar in cells treated with scrambled siRNA or siRNAs against TMEM132A, with the global protein glycosylation inhibitor Tunicamycin (Tun) treatment as a positive experimental control (Coombs et al., 2010) (Figure 6B).

WNT palmitoylation catalyzed by Porcupine (PORCN) in the ER occurs by the addition of the cis-Δ9-monounsaturated fatty acid palmitoleate (C16:1Δ9) (Komekado et al., 2007; Kurayoshi et al., 2007), which is generated from the saturated fatty acid palmitate (C16:0) by the ER resident protein stearoyl-CoA desaturase (SCD) (Rios-Esteves and Resh, 2013), onto a conserved serine (at Ser209 site of WNT3A) via an oxyester bond (*O*-palmitoylation) and plays crucial role in WNT trafficking between membrane compartments (Coombs et al., 2010; Gao and Hannoush, 2014b). Insufficient Wnt ligand palmitoylation upon PORCN repression affects Wnt ligand binding with WLS, as well as ERAD of WLS (Coombs et al., 2010; Glaeser et al., 2018). Here we used metabolic incorporation of fatty acid ortholog palmitic acid alkyne (Alk-C16:0), combined with IP and click chemistry (with azide-tagged biotin) to determine WNT3A and WNT5A palmitoylation levels. However, no obvious change was found when knocking down TMEM132A in HEK 293T Cells (Figure 6C). Together these results indicate that TMEM132A does not play a role in these post-translational modifications of Wnt ligand.

Wnt ligand binding to WLS is the crucial step for the trafficking of WNT cargo through the secretory pathway. Although Wnt ligand palmitoylation, which is required for WLS-WNT binding was not altered by TMEM132A knockdown, we still questioned and investigated whether TMEM132A functions in WLS–Wnt ligand interaction in another unknown manner. Co-IP experiment was performed using transient transfected HEK 293T cells to evaluate whether the binding affinity of WNT3A and WNT5A to WLS was impacted by the addition of TMEM132A. As expected, FL Myc-tagged WLS (WLS-Myc) can co-immunoprecipitate both WNT3A and WNT5A, as well as TMEM132A. Notably, the addition of TMEM132A robustly increases the binding of WLS with both WNT3A and WNT5A (Figure 6D). These data indicate that TMEM132A significantly enhances the interaction between WLS and Wnt ligand.

The fact that aa 1–264 of WLS, which contains the Wnt ligand binding domain, strongly interacts with TMEM132A (Figure 3G), raises the possibility that there might also be an interaction between TMEM132A and Wnt ligand. To test this hypothesis, co-IP experiments were performed in HEK 293T cells. Indeed, both WNT3A and WNT5A can be co-immunoprecipitated by TMEM132A (Figure 6E). This experiment is consistent with two possibilities: the interaction of TMEM132A with Wnt ligand is direct or through a trimeric complex with WLS.

## Discussion

**FIGURE 7:**
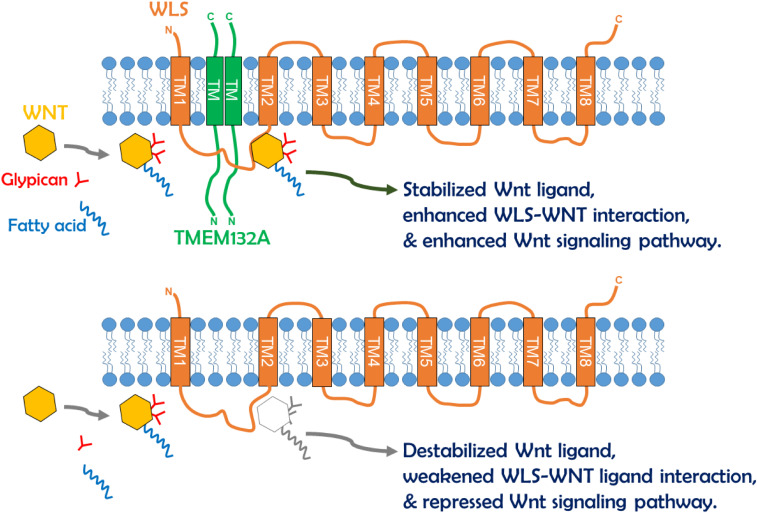
Schematic model of TMEM132A regulation of Wnt signaling pathway within signal sending cells. The TMEM132A homodimer (or oligomer) interacts with the Wnt ligand trafficking protein WLS to increase WNT binding and WNT stabilization, and to enhance WNT secretion and Wnt signaling pathway activation. In the absence of TMEM132A, Wnt ligand becomes unstable, and WLS–Wnt ligand interaction is weakened, leading to repressed Wnt signaling pathway.

Cell–cell communication by Wnt ligands controls a multitude of fundamental patterning processes during embryogenesis and tissue homeostasis in adults (Freese et al., 2010; Yang, 2012). Extensive regulatory mechanisms are encapsulated within this signaling pathway, and much is known in the context of Wnt signal receiving cells. However, the regulation of WLS-Wnt ligand trafficking in signal sending cells is less well understood. Here, we have uncovered a new factor in the control of the Wnt signaling pathway. The single-pass transmembrane protein TMEM132A plays a novel role in regulating the Wnt signaling pathway in signal producing cells. The *N*-terminus of TMEM132A acts as a self-interacting domain, and the TM domain provides a crucial compartment for interacting with WLS. TMEM132A stabilizes Wnt ligand and strongly enhances the WLS–Wnt ligand interaction necessary for WNT trafficking (schematic model in Figure 7). Given the fact that TMEM132A and WLS highly colocalize in the ER, vesicles and plasma membrane (Figure 2), TMEM132A likely functions in transporting WLS-Wnt ligand cargo from the ER/Golgi to the plasma membrane for efficient Wnt signal production.

Since TMEM132A and WLS are both transmembrane proteins, and the interaction occurs between the TM domain of TMEM132A and mainly aa 1–264 of WLS, it is likely that the interaction occurs within the membrane. This would suggest that the binding domain of WLS with TMEM132A localizes in either or both TM domains 1 and 2 of WLS. The Wnt ligand binding domain (aa 100–232 of WLS) overlaps with the TMEM132A binding domain, raising the possibility that there is a tripartite relationship between TMEM132A, WLS and Wnt ligand. This could explain why addition of TMEM132A increases WLS-WNT3A or WNT5A interaction (Figure 6D). We did find that WNT3A or WNT5A can be co-immunoprecipitated by TMEM132A (Figure 6E), however, it is unclear whether TMEM132A interacts with Wnt ligand directly or through the interaction with WLS, as the interaction we observed is not as strong as that of TMEM132A with WLS. Another possibility is that TMEM132A acts as a chaperone of WLS, to allow WLS to form an appropriate conformation for Wnt ligand accessibility.

Dysfunction of WLS, the exclusive carrier of Wnt ligands, causes retention and accumulation of Wnt ligand in the ER (Banziger et al., 2006; Das et al., 2015). The fact that we observed decreased levels of WNT3A or WNT5A upon TMEM132A depletion suggests this is not mainly due to interrupted WLS-Wnt ligand binding. We did not find a change in WNT3A or WNT5A glycosylation or palmitoylation status upon knockdown of TMEM132A in HEK 293T cells (Figures 6B,C), although improper post-translational modification of WNT protein triggers ER stress via UPR and ERAD (Ruggiano et al., 2014). Nevertheless, it was reported that loss of TMEM132A might increase ER stress and apoptosis induced by serum starvation (Oh-hashi et al., 2010). Here we have focused on TMEM132A/WLS/WNT interaction but there may be broader effects of TMEM132A depletion on global protein synthesis and susceptibility to ER stress. Nevertheless, we did not observe any obvious changes in cell viability or general ERAD induction upon knockdown of TMEM132A in normally growing cultured cells. Moreover, we did not observe significantly destabilized turbo RFP (Figure 4E) whereas we did see significantly decreased WNT3A or WNT5A protein, which may suggest functional specificity of TMEM132A for certain proteins. We also observed a decrease in the level of secreted WNT3A or WNT5A. This could in part reflect the decreased pool of WNT in the cytoplasm. However, our evidence that TMEM132A increases WLS–WNT interaction and that TMEM132A and WLS colocalize to ER, vesicles and plasma membrane suggest the TMEM132A is required for Wnt ligand trafficking and secretion. TMED6, a P24 family member, might interact with both TMEM132A and WLS, as predicted by BioGRID (Oughtred et al., 2019). The P24 proteins, a family of ∼24 kDa type I transmembrane proteins, play critical roles in bidirectional transport processes at the ER-Golgi interface (Strating and Martens, 2009). Hence, future studies should reveal whether TMEM132A might function in Wnt ligand export from the ER and/or shuttling to the Golgi through interactions with WLS and P24 family protein(s). Overall, our studies identify a novel protein interaction between TMEM132A and WLS which is necessary for WNT trafficking within the signal sending cells. Future studies of TMEM132A mutant mice will allow these biochemical and mechanistic findings to be explored in an *in vivo* context.

## Experimental Procedures

### Plasmid Construction

To obtain the mouse TMEM132A, WLS, WNT3A, and WNT5A coding sequences, total RNA was extracted from mouse tissue using TRIzol Reagent (Thermo Fisher Scientific, #15596018), and cDNA was generated by reverse transcription using SuperScript IV First-Strand Synthesis System (Thermo Fisher Scientific, #18091050) according to the manufacturer‘s instruction. Coding sequences without the stop codon were amplified: full-length (FL) TMEM132A (amino acids (aa) 1–1,018, according to NCBI NP_598565.2 protein sequence), WLS (aa 1–541, according to NCBI NP_080858.3 protein sequence), WNT3A (aa 1–352, according to NCBI NP_033548.1 protein sequence), and WNT5A (aa 1–380, according to NCBI NP_033550.2 protein sequence). Clones were Sanger sequenced and subcloned into SgfI/MluI restriction enzyme sites of pCMV6-AC-DDK/Myc/HA/GFP/RFP (Origene, #PS100005/ PS100003/ PS100004/ PS100010/ PS100034), respectively, according to the need of specific *C*-terminal tag. The FL TMEM132A contains the signal peptide (aa 1–32, abbreviated as S), *N*-terminus (aa 33–844, abbreviated as N), transmembrane domain (aa 845–867, abbreviated as T), and *C*-terminus (aa 868–1,018, abbreviated as C), thus was also named as SNTC. Based on this, truncations of SNT, STC, SN, C, SNC, and ST were constructed by subcloning corresponding sequences into SgfI/MluI restriction enzyme sites of pCMV6-AC-DDK. WLS truncations of aa 1–264, aa 265–331, aa 332–428, and aa 429–541 were constructed by subcloning corresponding sequences into SgfI/MluI restriction enzyme sites of pCMV6-AC-HA.

For mass spectrometry studies, the pSCDTG(P) control vector was obtained by introducing the Streptavidin binding peptide (MDEKTTGWRGGHVVEGLAGELEQLRARLEHHPQGQREPS GGCKLG, abbreviated as SBP), the Calmodulin binding peptide (KRRWKKNFIAVSAANRFKKISSSGAL, abbreviated as CBP), Flag tag (DYKDDDDK), and the T2A proteolytic site (GSGEGRGSLLTCGDVEENPGP) cassette at the *N*-terminus of turbo GFP in pCMV6-AC-GFP, and the original Neomycin resistance gene sequence was replaced with Puromycin resistance gene sequence. FL TMEM132A sequence without the stop codon was subcloned into SgfI/MluI restriction enzyme sites of pSCDTG(P) to obtain pSCDTG(P)-T132A expressing TMEM132A-SBP-CBP-Flag-T2A-turbo GFP fusion protein see schematic in Supplementary Materials.

Plasma membrane marker was made by subcloning the myristolyation/palmitoylation sequence from Lck tyrosine kinase (Sun et al., 2017) (ATGGGCTGTGTCTGCAGCTC AAACCCTGAA) into SgfI/MluI restriction enzyme sites of pCMV6-AC-RFP, named as PM-RFP. ER marker was constructed by subcloning the ER retention signal (KDEL) sequence from Human Calreticulin (AAGGACGAGCTGTAG) into XhoI/PmeI restriction enzyme sites of pCMV6-AC-RFP, named as ER-RFP.

### Cell Culture and Transfection

HEK 293T cells and HeLa cells were obtained from the American Type Culture Collection (ATCC, #CRL-3216 and #CCL-2) and cultured in high-glucose Dulbecco’s Modified Eagle Medium (DMEM, Thermo Fisher Scientific, #11995065) supplemented with 10% FBS (Thermo Fisher Scientific, #A3840001) at 37°C with 5% CO_2_. MEFs were isolated from E13.5 embryos dissected from a mating cross of HET male and female *Tmem132a^+/–^* mice (*Tmem132a*^*tm1b(KOMP)Wtsi*^, from UC Davis Knockout Mouse Project Repository). All animals were handled according to the protocol approved by the Institutional Animal Care and Use Committee (IACUC) at the University of Colorado Boulder. MEFs were cultured in DMEM supplemented with 10% FBS and 1% GlutaMAX Supplement (Thermo Fisher Scientific, #35050079) at 37°C with 5% CO_2_ and used within five passages.

Cells were seeded and maintained overnight to reach ∼80% confluency at the time of transfection. PEI MAX 40K (Polysciences, #24765-1) or Lipofectamine 3000 Transfection Reagent (Thermo Fisher Scientific, #L3000015) was used for plasmid transfection, and Lipofectamine RNAiMAX Reagent (Thermo Fisher Scientific, #13778-150) was used for siRNA transfection, following manufacturer’s protocol. The following Silencer Select siRNAs from Thermo Fisher Scientific were used: Negative control No. 1 siRNA (siCtrl, #4390843), human TMEM132A No.1 siRNA (si#1, #s29890) and human TMEM132A No. 2 siRNA (si#2, #s29892). When siRNA and plasmid were both used, siRNA (5 nM final concentration) was first transfected, and 24 hours later the plasmid was transfected.

### Sample Preparation for Mass Spectrometry

HEK 293T cells were transfected with pSCDTG(P) and pSCDTG(P)-T132A under Puromycin selection (2.5 μg/ml final concentration). Single colonies were isolated by cloning cylinder and expanded to obtain stable integration of the control and TMEM132A constructs. The cells were lysed, protein expression examined and subjected to TAP using InterPlay TAP purification kit (Agilent Technologies, #240107) according to the manufacturer‘s instruction. FASP was subsequently performed (Wisniewski et al., 2009) and the digested peptides were subjected to mass spectrometry which was processed and analyzed by the Mass Spectrometry Proteomics Shared Resource Facility at University of Colorado Anschutz Medical Campus. The data is provided in the Supplementary Materials with protein abundance expressed as reads. A higher number of reads indicates more hits and zero indicates no hit was found in specific group.

### Confocal Microscopy and Analysis

HeLa cells were seeded and transiently transfected when reaching ∼80% confluency. 24 hours post transfection, cells were trypsinized, counted and re-seeded on glass cover slips pre-coated with collagen I (Corning, #354265) at low confluency. Another 24 hours later, cells were fixed in 4% PFA in PBS for 15 min at room temperature. Fixed cells were permeabilized with 0.1% Triton x-100 in PBS for 15 min at room temperature and when necessary stained with α-Flag (MilliporeSigma, #F1804, produced in mouse) and α-HA (MilliporeSigma, #H6908, produced in rabbit) primary antibodies diluted in BlockAid Blocking Solution (Thermo Fisher Scientific, #B10710) at 4°C overnight. Cells were then incubated with Donkey α-Mouse IgG (H + L) Highly Cross-Adsorbed Secondary Antibody, Alexa Fluor Plus 488 (Thermo Fisher Scientific, #A32766) and Donkey α-Rabbit IgG (H + L) Highly Cross-Adsorbed Secondary Antibody, Alexa Fluor Plus 647 (Thermo Fisher Scientific, #A32795) diluted in BlockAid Blocking Solution for 2 hours at room temperature. The nuclei were counterstained with Hoechst (10 μg/ml final concentration in PBS) for 15 min at room temperature. Specimens was mounted using ProLong Glass Antifade Mountant (Thermo Fisher Scientific, #P36980) and captured using Nikon TiE Inverted Spinning Disk Confocal Microscope using 100×/1.45NA Plan Apo λ Oil WD 0.13 (mm) objective. Images were processed and analyzed using Fiji (NIH) software (Schindelin et al., 2012).

### Co-Culture and Dual-Luciferase Reporter Assay

Co-culture assay was performed as described previously (Banziger et al., 2006) with minor modification. Briefly, turbo GFP or WNT3A or WNT5A expressing plasmid were transfected into HEK 293T cells and Wnt signaling pathway reporter was transfected into wild type or *Tmem132a^–/–^* MEFs. 24 hours later, the cells were trypsinized, counted, mixed at 1:1 ratio of HEK 293T cells to MEFs and co-cultured for another 48 hours. Cells were lysed and subjected to dual-luciferase assay as described previously (Yu et al., 2016; Li et al., 2018). TCF/LEF responsive Topflash firefly luciferase reporter was used for evaluation of canonical Wnt/β-Catenin signaling activation. Activated c-Jun responsive pFR firefly luciferase reporter accompanied by pFA2-c-Jun was used for evaluation of non-canonical Wnt/PCP signaling activation (Shi et al., 2012, 2018). The constitutive Renilla luciferase reporter pGL4.74[*hRluc*/TK] (Promega, # E6921) served as an internal control.

### Real-Time Quantitative PCR

Total RNA extraction and cDNA synthesis were carried out as described above. Real-time quantitative PCR was performed on a Roche LC480 thermocycler using Luna Universal Probe qPCR Master Mix (NEB, #M3004X). Relative fold change in gene expression was calculated as described previously (Li et al., 2013a,b). Primers and Universal Probe Library (UPL, Roche Diagnostics) are as follows: Human *TMEM132A* F/R (+ UPL #1): CCCTGGACGTCGTGAGAG/GAAGTGTTCAGGGGCGT CTA; Human *GAPDH* F/R (+ UPL #45): TCCACTGGCGTCTT CACC/GGCAGAGATGATGACCCTTTT; Mouse *Axin2* F/R (+ UPL #50): CCATGACGGACAGTAGCGTA/GCCATTGG CCTTCACACT; Mouse *Ccnd1* F/R (+ UPL #1): CATC CATGCGGAAAATCG/CAGGCGGCTCTTCTTCAA; Mouse *Gapdh* F/R (+ UPL #77): GACAATGAATACGGCTACAG CA/GGCCTCTCTTGCTCAGTGTC.

### Western Blot, Immunoprecipitation and Click Chemistry

For Western blots without immunoprecipitation (IP)/co-IP purpose, cells were transfected with expression constructs as noted in the text or not treated. Whole cell lysates were prepared using modified RIPA buffer [50 mM Tris-HCl pH 8.0, 150 mM NaCl, 10% Glycerol (v/v), 1 mM EDTA, 1% NP-40 (v/v), 0.5% Sodium deoxycholate (w/v), 0.1% Sodium dodecyl sulfate (SDS) (v/v)], supplemented with proteinase inhibitor cocktail (1 μg/ml Aprotinin, 1 μg/ml Leupeptin, 1 μg/ml Pepstatin, 1 mM Na_3_VO_4_, 10 mM NaF). Cytoplasmic proteins were extracted for IP/co-IP experiments using n-Dodecyl-beta-Maltoside Detergent (DDM, Thermo Fisher Scientific, #89903) or NP-40 lysis buffer [(50 mM Tris-HCl pH 8.0, 150 mM NaCl, 10% Glycerol (v/v), 1 mM EDTA, 1% DDM or 0.5% NP-40 (v/v))], supplemented with proteinase inhibitor cocktail. α-Flag M2 Magnetic Beads (MilliporeSigma, #M8823), Pierce α-c-Myc Magnetic Beads (Themo Fisher Scientific, #88842) and Pierce α-HA Magnetic Beads (Themo Fisher Scientific, #88836) were used for IP/co-IP experiment. Antibodies for α-TMEM132A (Proteintech, #25301-1-AP), α-WLS (MilliporeSigma, #MABS87), α-GAPDH (MilliporeSigma, #G9545), α-Phospho-β-Catenin (Ser45) (CST, #9564), α-β-Catenin (CST, #8480), α-Flag (MilliporeSigma, #F1804), α-Myc (Thermo Fisher Scientific, #13-2500), α-HA (MilliporeSigma, #H6908) and α-turbo RFP (Origene, #TA150061) were used in Western blot.

For protein stabilization assay, 100 μg/ml (final concentration) of Cycloheximide (CHX, MilliporeSigma, #5.08739) was added to the culture medium 24 hours post transfection to block global protein synthesis, and cells were harvested at indicated time points after CHX treatment.

For protein glycosylation assay positive control, 0.5 and 1 μg/ml (final concentration) of Tunicamycin (Tun, MilliporeSigma, #654380) was added to the medium 24 hours post transfection to block global protein glycosylation, and cells were harvested after another 24 hours.

For protein palmitoylation assay, 100 μM alkynyl palmitic acid (final concentration) (Alk-C16:0, Click chemistry tools, #25362) was used for metabolic labeling 12 hours post plasmid transfection, and cells were harvested in another 12 hours after chemical addition. Cells were lysed in 100 mM sodium phosphate buffer (pH 7.4) containing 150 mM NaCl, 10% Glycerol (v/v), 1 mM EDTA, 0.5% NP-40 (v/v)) and proteinase inhibitor cocktail. IP and on-bead click chemistry reaction were performed according to the protocol adapted from previously described (Gao and Hannoush, 2014a; Tuladhar et al., 2019). Briefly, the reaction was performed in 100 mM sodium phosphate buffer (pH 7.4) containing 1 mM CuSO_4_ (MilliporeSigma, #451657-10G), 5 mM BTTAA (Click chemistry tools, #1236-100) and 15 mM sodium ascorbate (MilliporeSigma, #11140-50G) in the presence of 40 μM biotin azide plus (Click Chemistry Tools, #1488-5) for 1 h at room temperature with gentle agitation. IRDye 800CW Streptavidin (1:1,000 at 4°C overnight) (LI-COR, #926-32230) diluted in Intercept (TBS) Buffer (LI-COR, #927-60001) or α-biotin (HRP conjugate, 1:500 at 4°C overnight) (CST, #7075) diluted in 100 mM sodium phosphate buffer (pH 7.4) containing 150 mM NaCl, 0.1% Tween-20 (v/v), 0.4% Triton X-100 and 2.5% BSA was used to visualize palmitoylated WNT3A or WNT5A in Western blot.

All Western blots were imaged on a BIO-RAD ChemiDoc MP Imaging system and quantification was calculated by measuring band grayscale using Fiji (NIH) software (Schindelin et al., 2012). All original uncropped images for building Western blot figures are available in the “Original Western Blot Images” of Supplementary Materials. Non-specific bands (indicated by ^∗^) are specified as such when they appear in the empty vector transfected control groups at the same molecular weight in the same gel, and the band sizes are clearly different from the predicted size.

### Data Collection and Statistical Analysis

All experiments except for confocal microscopy, used samples were collected from at least three technical replicates and pooled to minimize sample variation. All experiments were performed at least twice and representative results are shown. For Wnt ligand and β-Catenin/Phospho-β-Catenin detection in MEFs, co-culture assay, and real-time quantitative PCR, at least three biological replicates were performed and data are presented as the mean ± S.D., and *p*-value was calculated by Student’s *t*-test and considered significant when less than 0.05.

## Data Availability Statement

The original contributions presented in the study are included in the article/Supplementary Material, further inquiries can be directed to the corresponding author.

## Ethics Statement

The animal study was reviewed and approved by Institutional Animal Care and Use Committee (IACUC) at the University of Colorado Boulder.

## Author Contributions

BL and LN designed the project and prepared the manuscript. BL performed the experiments. Both authors contributed to the article and approved the submitted version.

## Conflict of Interest

The authors declare that the research was conducted in the absence of any commercial or financial relationships that could be construed as a potential conflict of interest.

## Footnotes

1 https://www.mousephenotype.org/data/genes/MGI:2147810
