# Supplementary figures and images for: TMEM132A, a Novel Wnt Signaling Pathway Regulator Through Wntless (WLS) Interaction
Source: Front Cell Dev Biol. 2020 Nov 26;8:599890. doi: 10.3389/fcell.2020.599890 (PMC7726220; doi:10.3389/fcell.2020.599890)

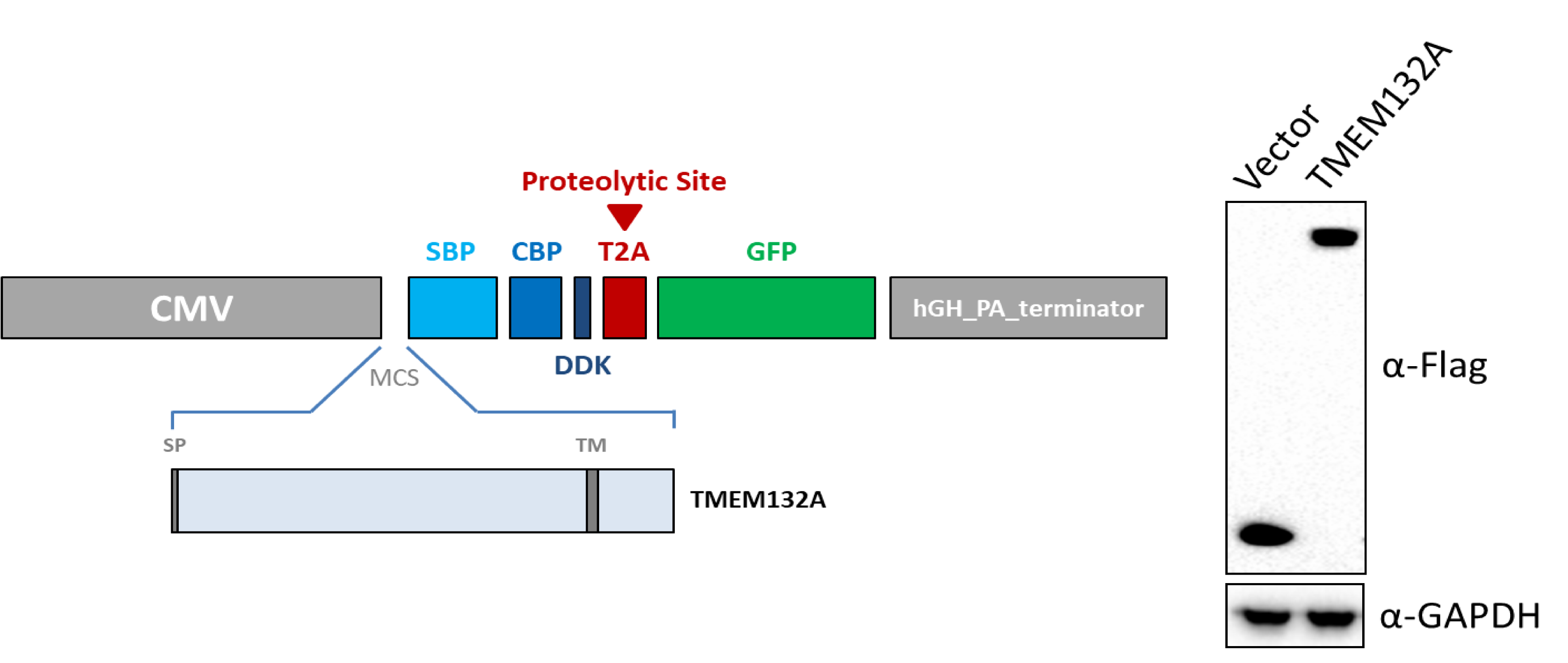

Supplement: Supplementary file 1 [file Image_1.tif]
